# Effect of orbital decompression surgery on the choroidal profile in patients with thyroid eye disease

**DOI:** 10.1038/s41598-024-65884-7

**Published:** 2024-06-28

**Authors:** Seyed Mohsen Rafizadeh, Ali Momeni, Masoud Rahimi, Hamid Riazi-Esfahani, Mehdi Yaseri, Hamidreza Ghanbari, Elias Khalili Pour

**Affiliations:** 1 Department of Orbital and Oculoplastic Surgery, Farabi Eye Hospital, Tehran University of Medical Sciences, Tehran, Iran; 2 Retina Service, Farabi Eye Hospital, Tehran University of Medical Sciences, South Kargar Street, Qazvin Square, Tehran, Iran; 3 Department of Biostatistics and Epidemiology, Tehran University of Medical Sciences, Tehran, Iran

**Keywords:** Thyroid eye disease (TED), Orbital decompression, Choroid vascularity index (CVI), Choroidal thickness, Eye diseases, Thyroid diseases

## Abstract

This study aimed to investigate the effect of orbital wall decompression surgery and reduction of proptosis on the choroidal vascularity index (CVI) and subfoveal choroidal thickness (SFCT) in patients with thyroid eye disease (TED). Fifty-one eyes from 38 patients with controlled TED and proptosis were enrolled in this study. The majority of the patients (50.9%) had a clinical activity score (CAS) of zero, and none had a CAS greater than 2. The patients underwent a complete baseline ophthalmologic examination, and their choroidal profile alterations were monitored using enhanced depth imaging optical coherence tomography (EDI-OCT) before and during the three months after surgery. Changes in SFCT, luminance area (LA), total choroidal area (TCA), and the choroidal vascularity index (CVI) were measured as the ratio of LA to TCA in EDI-OCT images. The participants had an average age of 46.47 years, and 22 were female (57.9%). The SFCT of the patients exhibited a significant reduction over the follow-up period, decreasing from 388 ± 103 to 355 ± 95 µm in the first month (p < 0.001) and further decreasing to 342 ± 109 µm by the third month compared to baseline (p < 0.001). The CVI exhibited a drop from 0.685 ± 0.037 at baseline to 0.682 ± 0.035 and 0.675 ± 0.030 at 1 and 3 months post-surgery, respectively. However, these changes were not statistically significant, indicating comparable decreases in both LA and TCA. There was a significant correlation between improved proptosis and reduction in SFCT (p < 0.001) but not with CVI (p = 0.171). In conclusion, during the three months of follow-up following orbital wall decompression, CVI did not change, while SFCT reduced significantly. Additionally, SFCT was significantly correlated with proptosis reduction, whereas CVI was not.

## Introduction

Thyroid eye disease (TED) is a multifactorial autoimmune disorder leading to inflammation of the retrobulbar and orbital tissues^1^. In the context of retrobulbar inflammation, proptosis arises from the congestion and fibrosis of surrounding tissues. Additionally, the hypertrophy of muscles and fat contributes to the protrusion of the eye^2^. It is more common in women between the ages of thirty and fifty but is more severe in men^3,4^. Although TED is more commonly associated with hyperthyroidism (Graves’ disease), it can also occur in euthyroid or hypothyroidism states^1^. Literature proves that smoking, late-onset hyperthyroidism (over fifty years old), prolonged disease, and poor control of thyroid disease are correlated with TED^4,5^.

TED has a vast range of manifestations, from mild proptosis to sight-threatening conditions such as dysthyroid optic neuropathy (DON)^6^. The treatment of TED depends on the severity and activity of the disease. In the active phase, medical treatment with corticosteroids, immunomodulators, and orbital radiotherapy is recommended. Surgical treatment is usually not recommended in the active phase of the disease. After the inflammation is resolved with medical treatments, orbital decompression surgery can be performed to reduce the patient's proptosis^7^.

Orbital decompression may be necessary in advanced sight-threatening cases (exposure keratopathy, DON, or elevated intraocular pressure) to alleviate intraorbital pressure and reduce compression on vital structures such as the optic nerve and ophthalmic veins^2,8^. With this invasive surgery, the surgeon ablates the medial wall, the lateral wall, the orbital floor, or more than one orbital wall with or without fatty tissue, depending on the severity of the disease^8^.

The fact that the choroid receives more than 70% of the ocular blood flow is widely acknowledged, and its profile can undergo variations in various inflammatory conditions such as TED^9^. Moreover, studies have demonstrated changes in ocular blood flow in individuals with TED^10,11^. Subfoveal Choroidal Thickness (SFCT) and Choroidal Vascularity Index (CVI) are two crucial parameters employed in the analysis of the choroid within optical coherence tomography (OCT) images^12,13^. Both metrics prove valuable in studying the choroid, but they carry distinct clinical implications and varying degrees of significance. SFCT, measured by OCT, represents the distance between the posterior edge of the retinal pigment epithelium (RPE) and the choroid-sclera interface^14^. The CVI is a relatively recent and more sensitive metric that provides insights into choroidal vascular anatomy. Its assessment involves binarization techniques applied to OCT images, defining it as the ratio of the luminal area (LA) to the total choroidal area (TCA)^12,13^.

An association has been identified between the activity of TED, and both the increase in SFCT and the elevation of CVI. These findings indicate the possibility of using enhanced depth imaging optical coherence tomography (EDI-OCT) to measure CVI/SFCT as an objective clinical marker for TED activity assessment^15,16^. Despite existing such evidences, the alterations of the choroidal profile linked to the relief of orbital pressure after orbital wall decompression remain uncertain.

In this study, our aim was to investigate the effect of orbital wall decompression surgery on the choroidal profile using EDI-OCT imaging. Additionally, we explored the alterations in SFCT and CVI and their correlation with the decrease in proptosis over a 3-month follow-up period.

## Methods

This study was a prospective interventional case series conducted at Farabi Eye Hospital from January 2021 to January 2023 in patients with TED and 20–30 mm proptosis. The study protocol adhered to the tenets of the Declaration of Helsinki and was approved by the local institutional review board (IR.TUMS.FARABIH.REC.1401.017). All participants provided written informed consent before participating in the study.

The patients underwent a thorough, complete ophthalmologic examination, including a clinical history, slit-lamp biomicroscopy, and indirect dilated ophthalmoscopy. Their demographic data along with their previous medical treatment and comorbidities, results of ophthalmologic examinations, and clinical activity score (CAS) of TED were recorded. We used a Hertel exophthalmometer to measure the amount of proptosis in patients at baseline and during follow-up. With regard to the literature, the normal exophthalmometry value was considered to be 20 mm maximum^17^.

With respect to the literature, some parameters were considered to calculate the CAS, such as (1) retrobulbar pain at rest, (2) pain on eye movement when looking up and down, (3) eyelid edema, (4) eyelid erythema, (5) conjunctival hyperemia and injection, (6) conjunctival chemosis, and (7) inflammation of the plica semilunaris or caruncle. One score was assigned to each of these parameters^18^. A masked optometrist measured best corrected visual acuity (BCVA) using the Snellen chart at baseline and during the follow-up, and the results were converted to the logarithm of the minimum angle of resolution (LogMAR).

In this study, patients with thyroid ophthalmopathy who had bilateral proptosis with eye protrusion of at least 21 mm (in Hertel exophthalmometry) or unilateral proptosis with a proptosis of at least 2 mm compared to the normal eye were included. The exclusion criteria were patients under the age of 20 or above 65, any previous orbital surgery including orbital decompression, patients with optic nerve dysfunction, or exposure keratopathy, other retinal or choroidal diseases such as choroid neovascularization (CNV), diabetic macular edema (DME), history of panretinal photocoagulation (PRP) and so on, eyes with refractive error (spherical equivalent (SE)) with a range of − 4 to + 0.75 diopters, those with poor quality EDI-OCT images, patients who had active TED (if the total clinical activity score (CAS) was ≥ 3), and patients with uncontrolled thyroid function test.

### Intervention

To decompress the medial orbital wall, a medial transcaruncular orbitotomy was performed. After orbitotomy, periorbita was incised and the medial orbital wall was exposed with a periosteal elevator. The bone was then removed posterior to the posterior lacrimal crest and inferior to the ethmoidal arteries. Then the conjunctiva was repaired with an 8–0 Vicryl suture.

After medial decompression, an inferior transconjunctival orbitotomy was performed in the same session in patients who had severe proptosis and required double-wall decompression. After accessing the orbital floor bone, the medial portion of the orbital floor was removed medial to the infraorbital nerve canal, which extended posteriorly to the posterior wall of the maxillary sinus. Finally, the conjunctiva was repaired with an 8–0 Vicryl suture and the eye was bandaged with erythromycin ointment.

The dressing was removed the day after surgery and the patient was discharged. The patient was visited in the first week, the first month, and the third month after surgery. At each visit, a complete ophthalmologic examination was performed.

### Imaging

To evaluate the SFCT and CVI, patients underwent EDI-OCT using SD-OCT (Spectralis, Heidelberg Engineering, Heidelberg, Germany) at baseline, 1 month, and 3 months after decompression. The fovea-centered OCT B-scan raster pattern was captured after the patients were positioned appropriately. In brief, the scan speed was 40,000 A-scans and the axial resolution was 3.5 microns. A customized scan acquisition procedure with up to 19 raster lines of 9 mm scan length and 1536 A-scans per line was used to take the images. Using TruTrack Active Eye Tracking (Heidelberg Engineering, Heidelberg, Germany) for real-time eye tracking, 100 images were chosen as the automatic real-time image average. Only precisely centered, complete scans devoid of blink or motion artifacts and with a signal strength (Q score) of at least 20 were accepted^19^. The scans were performed between 9:00 and 11:00 am to avoid diurnal variations^20^. After each evaluation, two masked, independent reviewers judged the best image on a computer screen. If the two reviewers determined that both the inner and outer margins of the choroid were recognizable, the image was recorded and used for analysis.

Subfoveal choroidal thickness (SFCT), denoting the distance between the outer edge of Bruch's membrane-RPE complex and the innermost border of the choroidoscleral junction in the central subfoveal region, was manually measured utilizing the built-in calipers within the OCT software. The distinguishable central foveal horizontal B-scan on EDI-OCT was chosen to calculate CVI using Sonoda's Method^13^. CVI was measured using FIJI (an enhanced version of ImageJ software, version 1.51 h; National Institutes of Health, Bethesda, Maryland; accessible at http://imagej.nih.gov/Fiji/) according to the method described in the literature^12,21^. In summary, at first, ImageJ software was launched, and the EDI-OCT image opened; then, the polygon tool was used to determine the region of interest (ROI) along the entire length of the OCT scan. The upper border of the ROI was drawn along the basal edge of the RPE and the lower border along the choroidoscleral border to define the total choroidal area (TCA). Then, three choroidal vessels with lumens larger than 100 m were chosen randomly using the oval selection tool on the interface, and the software determined the average reflectivity of these regions. To minimize disturbance in the OCT image, the minimum value for the average luminance was set to zero. The image was converted into eight bits to obtain a binarized image and perform automatic local thresholding (Niblack method) on the binarized image^22^. There are several issues with the image processing methods used to determine CVI that need to be fixed. Visibility issues arise when retinal blood vessels are present because they might cover up the choroid in posterior segment OCT imaging. Algorithms are used by many OCT machines to "flatten" pictures so they fit better on report sheets, although this might distort the anatomy and have an impact on the CVI calculation. Furthermore, there is a possibility that the binary representation of the image, with its black and white pixels, may not precisely portray the stromal and vascular regions^23^. Even though the modified threshold is what determines the CVI, we have taken steps to lessen its impact so that the accuracy and consistency of our findings are preserved. This approach ensures uniformity across all samples analyzed in our research. We have chosen to apply Niblack's auto-local thresholding process, which has been shown by Agrawal et al. to be less sensitive to external and internal factors and to be more accurate^12^. It allows for a robust threshold selection process by integrating the average and deviation of every pixel inside the designated zone. First, the picture is transformed into binary form using Niblack's auto-local thresholding technique, which yields a clear representation of the choroid-scleral interface. Compared to other methods where the polygonal region is chosen before image binarization, our approach enables a more accurate delineation of the subfoveal choroidal area^13^. In our previously published study, we utilized the absolute agreement model of the inter-class correlation coefficient (ICC) on a series of twenty EDI-OCT images to evaluate the consistency between two investigators in CVI measurement and calculation. The ICC value obtained was 0.969, signifying a strong agreement between the investigators^24^.

Secondly, the final image was again converted into red, green, and blue colors to select the dark pixels that define the luminance area (LA). The LA was calculated as the sum of the dark pixel areas. The ratio of LA to TCA is referred to here as CVI. All manual segmentations, encompassing the delineation of RPE and sclerochoroidal boundaries, were executed by a proficient grader (AM) and independently verified by a second grader (EKP). In instances of disagreement, consensus was reached for the segmentation contours. All the SFCT and CVI measurements were repeated 1 and 3 months after decompression on the registered central foveal B-scan cut.

### Statistical analysis

To present data we used mean, standard deviation(SD), median, and range. For comparing parameters across various follow-up times, we adopted a generalized estimating equation (GEE) model. This approach allowed us to account for both the bilateral nature of some subjects' measurements and the correlation between measurements taken at different follow-up times. Additionally, we employed another GEE model to assess how baseline variables influenced parameter changes over time. This involved examining interaction terms between time and these baseline variables to better understand their relationship with parameter changes across different follow-up periods. All statistical analysis performed by SPSS software (IBM Corp. Released 2019. IBM SPSS Statistics for Windows, Version 26.0. Armonk, NY: IBM Corp.). A P-value less than 0.05 was considered statistically significant.

### Ethics declarations

This study has been approved by the local institutional review board of Tehran University of Medical Sciences IR.TUMS.FARABIH.REC.1401.017. The study was performed in accordance with the Helsinki Declaration of 1964, and its later amendments.

### Consent to participate/consent to publish

All patients provided informed consent to participate in the study and publish the results.

## Results

This research involved an evaluation of 51 eyes from 38 patients, of which 13 were bilateral and 22 were female (57.9%). The participants' average age was 46.47 years, with a range of 24 to 64 years. Twenty-one patients (55.3%) were found to be younger than 51 years of age, whereas seventeen patients (44.7%) were 51 years of age or older. All of the participants exhibited TED in a remission phase. A total of 31 eyes (61%) underwent medial decompression, while 20 eyes (39%) underwent both inferior and medial decompression. The majority of the patients in this study had a CAS of 0 (50.9%), and all of them had a score below 3, indicating they were in the inactive phase. A summary of demographic information, intervention categories, and CAS is provided in Table 1.

**Table 1:** The demographic data and the intervention type analysis in the cases.

	Count	Column N %
Age (Binned)		
< = 50	21	55.3%
51+	17	44.7%
Sex		
Male	16	42.1%
Female	22	57.9%
Surgery		
Medial	31	60.8%
Inferior + Medial	20	39.2%
CAS		
0	26	50.9%
1	17	33.3%
2	8	15.8%

The patients had a spherical equivalent (SE) range between − 4 and + 0.75, with a mean of − 0.75 and a median of − 0.50. The preoperative proptosis measured 24.2 ± 2.6 mm, ranging from a minimum of 20 mm to a maximum of 30 mm. The baseline best-corrected visual acuity (BCVA) for the patients was 0.11 ± 0.22 LogMAR. The subfoveal choroidal thickness (SFCT) and choroidal vascularity index (CVI) values at baseline were 388 ± 103 µm and 68.5 ± 3.7, respectively.

Alterations in proptosis, and choroidal parameters at one and three months following treatment are detailed in Table 2.

**Table 2:** Patients examinations results during follow-ups in first and third month.

Parameter	Time	Mean ± SD	Change from baseline			
			Mean ± SD	95% CI		P‡
				Lower	Upper	
Proptosis	Baseline	24.2 ± 2.6				
	Month 1	21.9 ± 2.3	2.14 ± 1.17	1.8	2.48	< 0.001
	Month 3	20.7 ± 2	2.83 ± 1.41	2.32	3.34	< 0.001
CVI	Baseline	0.685 ± 0.037				
	Month 1	0.682 ± 0.035	0.31 ± 3.44	-0.72	1.34	0.945
	Month 3	0.675 ± 0.03	0.78 ± 3.47	-0.54	2.1	0.406
SFCT	Baseline	388 ± 103				
	Month 1	355 ± 95	23.52 ± 24.03	16.3	30.74	< 0.001
	Month 3	342 ± 109	43.35 ± 45.78	25.93	60.76	< 0.001

*CVI* Choroidal vascular index, *SFCT* Subfoveal choroidal thickness.

^‡^Using Generalized Estimating Equation (GEE) model.

The proptosis significantly decreased at one and three months following decompression (p < 0.001 for both measurements). Measurements were 24.2 ± 2.6 mm at baseline, 21.9 ± 2.3 mm at month 1, and 20.7 ± 2 mm at month 3.

**Figure 1:**
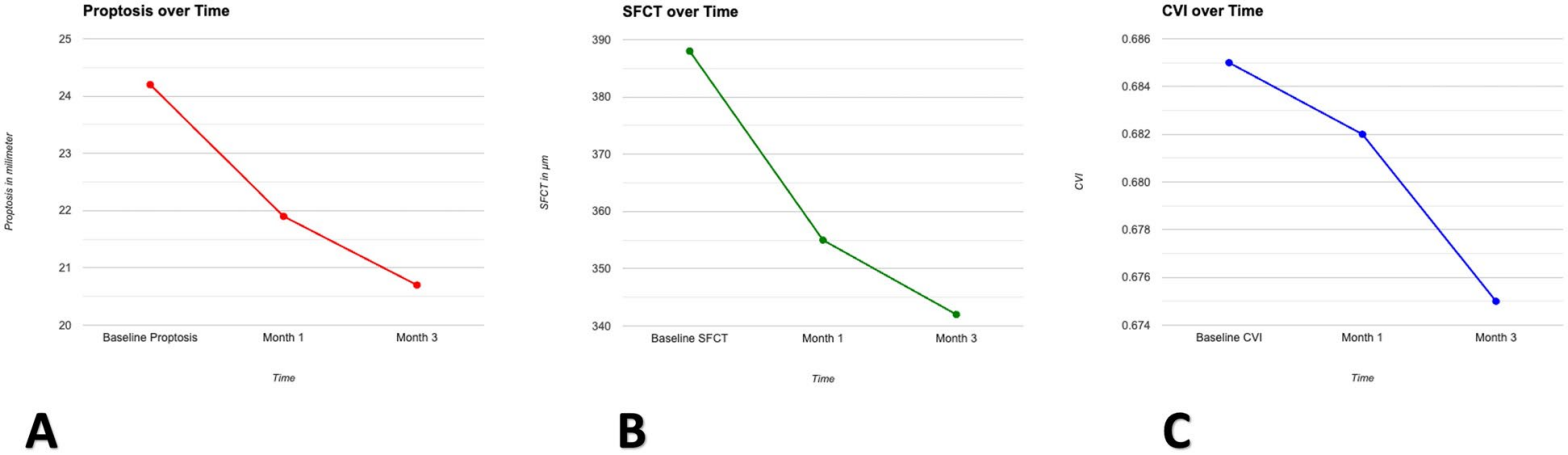
Line graphs of the parameters change during follow-up. The figure shows the mean proptosis changes (**A**), the mean subfoveal choroidal thickness (SFCT) changes (**B**), and the mean choroidal vascularity index (CVI) changes (**C**) over time during the study.

**Figure 2:**
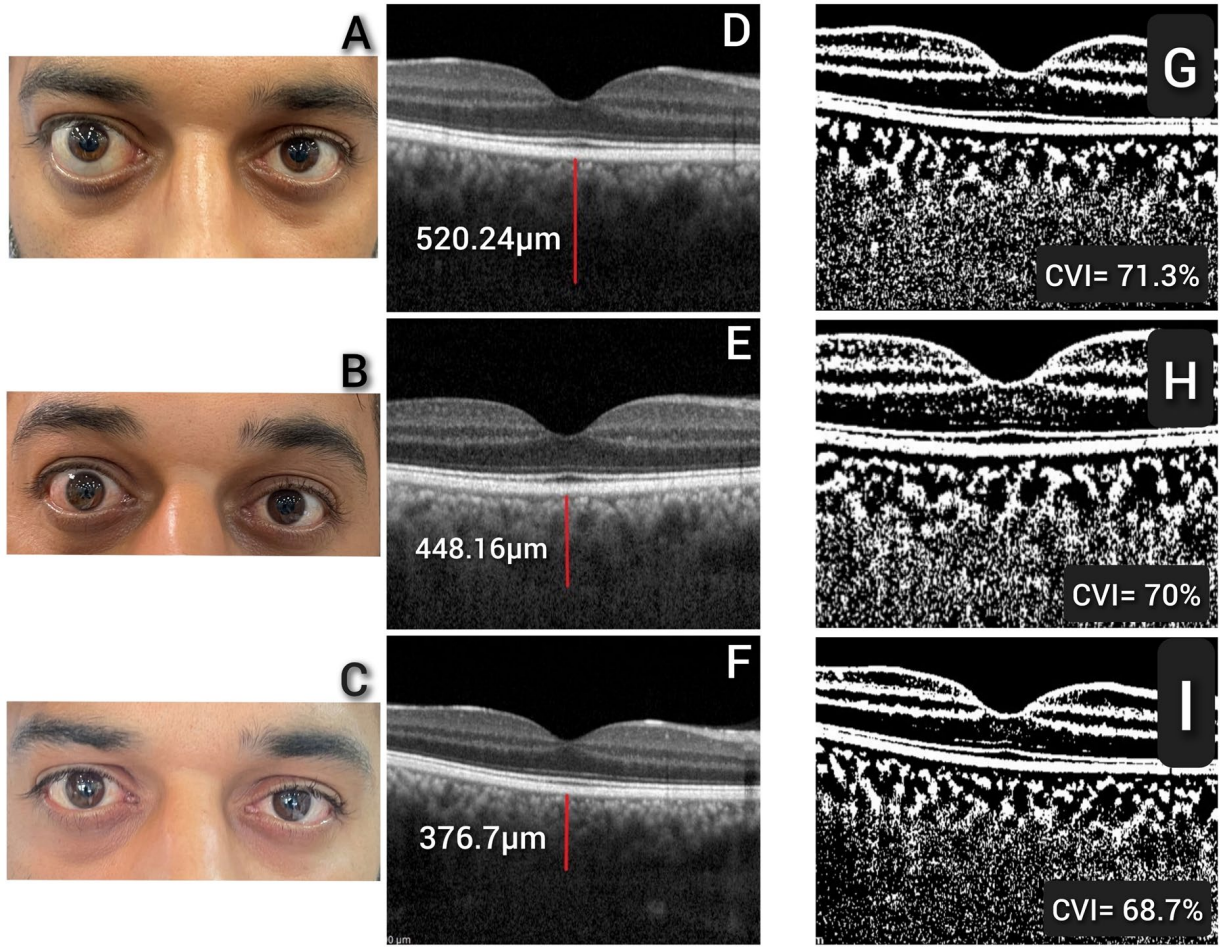
Improvement of proptosis and reduction of the subfoveal choroidal thickness in a patient with thyroid eye disease who underwent decompression of inferomedial orbital walls and fat of the right eye. (**A**) Pre-operation picture of the patient’s eyes. (**B**) Improvement of the proptosis after 1 month. (**C**) Improvement of the proptosis and inflammation after 3 months. (**D**) Pre-operation optical coherence tomography (OCT) of the right eye which shows the subfoveal choroid has a thickness of 520.24 μm. (**E**) Reduction of the subfoveal choroidal thickness after 1 months in OCT image (448.16 μm). (**F**) Reduction of subfoveal choroidal thickness after 3 months in the patient (376.7 μm). (**G**) Binarized OCT image which shows the amount of CVI before operation. (**H**) Binarized OCT image with CVI after 1 month. (**I**) Binarized OCT image with CVI after 3 months.

The SFCT decreased significantly from 388 ± 103 to 355 ± 95 µm within the initial month (p < 0.001 for change from baseline). Subsequently, by the third month, the thickness had further diminished to 342 ± 109 µm (p < 0.001 for change from baseline) (Figs. 1 and 2).

The CVI exhibited a decline from 0.685 ± 0.037 at baseline to 0.682 ± 0.035 and 0.675 ± 0.030 one and three months following the surgery, respectively. However, these changes failed to reach statistical significance at either the one-month (p = 0.945) or three-month (p = 0.406) follow-up (Figs. 1 and 2). As demonstrated in Table 1 of the supplementary file, the results of the study remained unaffected by the influence of the aforementioned parameters, as determined by multivariable analysis.

The correlation between changes in CVI and SFCT and the postoperative reduction in proptosis was investigated. SFCT and proptosis were the only variables showing a significant correlation. A decrease in SFCT was associated with a substantial improvement in proptosis (B = 8.43, 95% CI 4.19–12.67, p < 0.001). However, the mean postoperative proptosis change was not significantly associated with CVI changes (B = 0.17, 95% CI − 0.04 to 0.40, p = 0.117).

Although a more notable reduction in proptosis was observed in patients who underwent 2-wall decompression, with a borderline p-value of 0.051, there were no statistically significant differences in SFCT and CVI changes between patients who underwent one-wall and two-wall decompression (p = 0.475 and p = 0.790, respectively).

Although this study used GEE model analysis to determine the difference between the patients who underwent unilateral decompression and bilateral decompression, we used a separate analysis to ensure there was no statistical difference between them and the results were the same as the GEE model analysis which is shown in Table 2 in supplement file.

## Discussion

The current study investigated the pattern of modification of choroidal structure following orbital decompression, using EDI-OCT in patients with TED. The majority of research indicates an increase in choroidal thickness and CVI among individuals with TED compared to those without the condition, particularly in the subfoveal area. Moreover, various studies have noted heightened thickness in patients with active TED compared to those with inactive TED, although the findings are inconclusive. Lastly, numerous studies have established connections between increased choroidal thickness and deteriorated clinical assessments of disease activity, including the Clinical Activity Score (CAS)^15,25–29^. In this present investigation, we demonstrated a significant decrease in SFCT at both 1 and 3 months following orbital decompression. However, the reduction in CVI during this timeframe did not reach statistical significance. Interestingly, we found a notable correlation between the decrease in SFCT and the degree of proptosis improvement. Conversely, we observed no correlation between the alteration in CVI and the improvement in proptosis.

In recent times, numerous investigations have explored the influence of TED on choroidal thickness and CVI. One plausible pathophysiologic mechanism suggests that the heightened intraorbital pressure during the inflammatory stage of TED results in congestion of the superior orbital vein due to impaired drainage. This congestion, in turn, causes an increase in blood volume in the choroid, leading to thickening^25,26,29^. Notably, small-scale studies have indicated that orbital decompression surgery resolves pre-operative choroidal perfusion defects, although no study has specifically addressed choroidal thickness and CVI^30^.

We showed a significant reduction in SFCT after orbital decompression in patients with TED. While the exact mechanism responsible for the heightened choroidal thickness in individuals with TED remains unclear, the most probable cause is associated with the expansion of extraocular muscle volume involving tissue proliferation and edema^31^. Also, the other mechanisms contributing to the pathogenesis of TED include the production of cytokines and inflammation, both of which can result in orbital adipose tissue expansion. Histologic examinations of tissue structure have revealed the infiltration of glycosaminoglycans, lymphocytes, and immune complexes in extraocular muscle fibers and orbital adipose tissue. This infiltration results in edema, expansion of soft tissue, augmented intraorbital volume, and consequently elevated intraorbital pressure^32^. Specifically, the vascularity of the choroid makes it particularly susceptible to compromised vascular outflow and increased venous congestion due to heightened intraorbital pressures^15^. Consequently, choroidal thickness has been suggested as a potential imaging marker reflecting orbital congestion and high pressure in TED^33,34^. Alp and collaborators observed heightened orbital venous congestion and reduced flow velocity in the superior ophthalmic vein among individuals with TED when compared to healthy control subjects^35^. Otto et al. discovered that individuals with TED exhibited significantly elevated intraorbital pressure prior to surgical decompression. Following the surgery, the pressure was reduced by an average of 10 mmHg^36^. Nik and colleagues, utilizing high-speed indocyanine green angiography, demonstrated that orbital decompression could enhance choroidal circulation and improve blood congestion^30^. We posited that the decrease in orbital pressure following decompression could enhance the resolution of venous congestions by facilitating increased drainage through the vortex veins, consequently resulting in a reduction in subfoveal choroidal thickness (SFCT).

Utilizing EDI-OCT facilitates the assessment of CVI, providing a means to quantify both choroidal stromal and vascular components. Due to its independence from age, gender, refractive error, and intraocular pressure (IOP), CVI is becoming increasingly favored as a parameter for evaluating choroidal vascularity^13,37^. An elevation in CVI can stem from either an enlargement in the diameter or an increase in the number of choroidal blood vessels^12^. While the reduction in CVI following orbital decompression did occur, it did not reach statistical significance. Given the significant decrease in SFCT, it suggests that the concurrent reduction in the ratio of choroidal luminal and stromal areas may be proportional. In a study conducted by Yeter et al., it was discovered that the proportional increments in the luminal and stromal areas of the choroid in patients with TED were approximately equal. The closely correlated increases in luminal area (LA) and total choroidal area (TCA) might contribute to the absence of a significant difference in CVI between the TED and control groups in their study^26^. It can be conjectured that, in addition to vascular changes like vascular engorgement and enlargement of choroidal vessel diameters, certain modifications may also take place in the choroidal stroma in TED. It remains uncertain whether these stromal alterations occur as a compensatory response to vascular congestion or if there are specific structural changes in the stroma due to the direct effects of TED disease. The capillaries within the choroid possess fenestrations, particularly large pores that exhibit high permeability to low-molecular-weight substances like albumin and myoglobin^38^. It has been demonstrated that the choriocapillaris' elevated protein permeability facilitates the creation of a high oncotic pressure, likely playing a role in the movement of fluid out of the vessels through the choroidal stroma^39^. Hence, in alignment with the decrease in choroidal vessel engorgement following decompression, the fluid movement to the choroidal stroma may be diminished, resulting in a nonsignificant alteration in CVI after orbital decompression.

The rise in choroidal stroma components could be attributed to an increased synthesis of glycosaminoglycans (GAGs), triggered by the activation of stromal fibroblasts in the choroid due to circulating autoantibodies, as observed in the expansion of orbital tissue in patients with TED^40^. It is conceivable that the excessive production of GAGs, activated by orbital and choroidal fibroblasts, may concurrently increase the stromal area directly and the luminal area indirectly through venous congestion^26^.

Some studies in autoimmune conditions, such as lupus, have suggested that intense inflammation might lead to significant choroidal vasodilatation and stromal edema, even in patients without ocular involvement^41^. Recently, Evereklioglu et al. in a series of patients with Behçet disease showed that, although ocular inflammation can affect CVI, systemic inflammation without ocular involvement does not affect it^42^. The majority of the patients in this study had a CAS of 0, and all of them had a score below 3, indicating they were in the inactive phase with low intraocular inflammation which led to minimal CVI changes after orbital decompression.

In a multiple regression analysis conducted by Vivek Dave and colleagues, it was revealed that the CAS had a positive impact on CVI. The study demonstrated that CVI is effective in distinguishing between active and inactive TED eyes, with patients having lower CAS (indicating inactive TED) exhibiting lower CVI^16^. Since all the participants had well-managed TED with CAS scores lower than 3 in this study, the alteration in CVI was minimal following decompression.

Proptosis is another parameter that our study showed has a significant relation with SFCT, in other words, a decrease in proptosis led to a significant diminishment in SFCT. We also observed that there was no significant difference in CVI and CT changes between the patients that underwent one and two-wall decompression, this outcome could be influenced by a selection bias, as patients with more pronounced proptosis typically undergo a two-wall decompression^43^.

Our study has several limitations. Firstly, the relatively small sample size and the brief follow-up duration might impact the results. Secondly, we did not address the alterations in the systemic health status of participants. However, significant changes are improbable within the relatively short-term follow-up period. Thirdly, the optimal approach for evaluating CVI in both normal and pathological conditions involve a volumetric analysis of choroidal vascularity changes in the macular region. In our current study, all CVI assessments were limited to specific selected foveal horizontal B-scans. Agrawal et al.^12^ demonstrated that a single scan can effectively depict choroidal vascularity across the entire posterior pole in healthy individuals. Goud et al. evaluated the topographical variation of CVI in a three-dimensional macular area based on the ETDRS grid and indicated no notable variances among various rings, subfields, and quadrants of the grid^44^. The extrapolation of our results to assess changes in choroidal metrics in selected foveal slabs to the entire macular area in patients with TED should be approached with caution. The choroidal vascularity index (CVI) is often less impacted by physiological parameters than SFCT^12^. However, it is crucial to note that the influence of external variables like alcohol consumption, smoking, medications, and occupational exposures cannot be completely controlled and may still affect our results. Unlike SFCT, CVI remains unaltered by factors such as spherical equivalent (SE) and axial length (AL). Hence, CVI emerges as a valuable parameter for studying choroidal changes in different refractive errors^45^. However, in this study only patients who had a SE range of − 4 and + 0.75 were recruited to diminish this effect as possible. With such inclusion criteria, the effect of lateral magnification would also be mitigated. Although some inactive TED patients do not have increased inflammation symptoms, they show signs of increased orbital congestion. These signs, such as dilation or engorgement of the episcleral vessels and an increase in IOP (especially when looking up), are caused by increased intraorbital pressure. This increase in pressure reduces venous drainage and causes changes in the choroidal profile. Orbital decompression surgery expands the orbital space into the sinuses. As a result, reducing the pressure exerted on the vessels inside the orbit can cause regression of changes in the choroid profile. By following these changes during the months after orbital decompression as an objective measure, the effectiveness of orbital decompression can be checked better and more accurately. Finally, although the present study identified certain alterations in choroidal profiles following decompression in TED patients, further controlled prospective investigations are required to ascertain the clinical significance of these findings in patient management. Despite the mentioned limitations, to the best of our knowledge, this study is the first to assess the impact of orbital decompression on choroidal profiles in individuals with TED.

## Conclusion

In conclusion, the study unveiled a significant reduction in SFCT at both 1 and 3 months following orbital decompression (medial or inferomedial) in patients with TED. However, the decrease in CVI during this period did not reach statistical significance. Notably, a significant correlation was observed between the decrease in SFCT and the degree of proptosis improvement. Conversely, no discernible correlation was found between the changes in CVI and the improvement in proptosis.

### Supplementary Information

Supplementary Information.

## Data Availability

The datasets generated during and/or analyzed during the current study are available from the corresponding author on reasonable request.
