# Supplementary material for: Effect of orbital decompression surgery on the choroidal profile in patients with thyroid eye disease
Source: Sci Rep. 2024 Jun 28;14:14948. doi: 10.1038/s41598-024-65884-7 (PMC11213849; doi:10.1038/s41598-024-65884-7)
Supplement: Supplementary file 1 — Supplementary Information. [file 41598_2024_65884_MOESM1_ESM.docx]

***Supplement:***

Table 1. The effects of multivariable parameters in the results of the study show that the results were not affected by these parameters.

| Parameter | B | Std. Error | 95% Wald Confidence Interval | | |  |
| --- | --- | --- | --- | --- | --- | --- |
|  |  |  | Lower | Upper | P-value |  |
| 1^st^ month | 0.795 | 0.5816 | -0.345 | 1.935 | 0.17 |  |
| 3^rd^ month | 0.582 | 0.5642 | -0.523 | 1.688 | 0.30 |  |
| Baseline | Reference | . | . | . | . |  |
| Male | -1.063 | 1.5313 | -4.065 | 1.938 | 0.48 |  |
| Female | Reference | . | . | . | . |  |
| Medial decompression | -0.031 | 1.4861 | -2.944 | 2.882 | 0.98 |  |
| Inferomedial decompression | Reference | . | . | . | . |  |
| Age | -0.010 | 0.0389 | -0.086 | 0.066 | 0.80 |  |
| Proptosis | 0.234 | 0.1807 | -0.120 | 0.589 | 0.19 |  |
| MRD1 | 0.023 | 0.1398 | -0.251 | 0.297 | 0.86 |  |
| MRD2 | 0.406 | 0.4125 | -0.402 | 1.215 | 0.32 |  |
| Dependent Variable: CVI (Choroidal Vascular Index)  Model: Time, sex, surgery, age, Proptosis, Margin to reflex distance (MRD)1, MRD2 | | | | | | |
|  | | | | | | |

Table 2.

Analysis of the difference in the results of the study according to the unilateral and bilateral decompression shows no difference between the two groups.

| Parameter | B | Std. Error | 95% Wald Confidence Interval | | |
| --- | --- | --- | --- | --- | --- |
|  |  |  | Lower | Upper | P-value |
| CVI Baseline | 68.779 | 1.1081 | -2.807 | 1.536 | 0.556 |
| CVI 1^st^ month | -0.732 | 1.0720 | -2.834 | 1.369 | 0.494 |
| CVI 3^rd^ month | 1.195 | 1.2150 | -1.187 | 3.576 | 0.326 |
| SFCT pre | 20.414 | 34.1507 | -46.520 | 87.348 | 0.550 |
| SFCT 1^st^ month | -4.193 | 33.8831 | -70.602 | 62.217 | 0.902 |
| SFCT 3^rd^ month | 35.509 | 45.6152 | -53.895 | 124.913 | 0.436 |

Choroidal Vascular Index (CVI), Subfoveal choroidal thickness (SFCT)
